# MicroRNAs in Cardiac Autophagy: Small Molecules and Big Role

**DOI:** 10.3390/cells7080104

**Published:** 2018-08-11

**Authors:** Teng Sun, Meng-Yang Li, Pei-Feng Li, Ji-Min Cao

**Affiliations:** 1 Key Laboratory of Cellular Physiology, Ministry of Education, Department of Physiology, Shanxi Medical University, Taiyuan 030001, China; tengsun@qdu.edu.cn; 2 Institute for Translational Medicine, Qingdao University, Qingdao 266021, China; limengyang@qdu.edu.cn (M.-Y.L.); peifeng@ioz.ac.cn (P.-F.L.)

**Keywords:** microRNAs, autophagy, mitophagy, cardiac diseases, biomarker

## Abstract

Autophagy, which is an evolutionarily conserved process according to the lysosomal degradation of cellular components, plays a critical role in maintaining cell homeostasis. Autophagy and mitochondria autophagy (mitophagy) contribute to the preservation of cardiac homeostasis in physiological settings. However, impaired or excessive autophagy is related to a variety of diseases. Recently, a close link between autophagy and cardiac disorders, including myocardial infarction, cardiac hypertrophy, cardiomyopathy, cardiac fibrosis, and heart failure, has been demonstrated. MicroRNAs (miRNAs) are a class of small non-coding RNAs with a length of approximately 21–22 nucleotides (nt), which are distributed widely in viruses, plants, protists, and animals. They function in mediating the post-transcriptional gene silencing. A growing number of studies have demonstrated that miRNAs regulate cardiac autophagy by suppressing the expression of autophagy-related genes in a targeted manner, which are involved in the pathogenesis of heart diseases. This review summarizes the role of microRNAs in cardiac autophagy and related cardiac disorders. Furthermore, we mainly focused on the autophagy regulation pathways, which consisted of miRNAs and their targeted genes.

## 1. Overview of Autophagy and MicroRNAs

### 1.1. Autophagy

Autophagy is an evolutionarily conserved process of the lysosome-dependent degradation of cytoplasm components and damaged organelles, such as endoplasmic reticulum, peroxisomes, and mitochondria [1]. Factors that induce cellular senility and stress, including infections, toxics, hypoxia, and nutrient starvation, can induce autophagy, which plays a role in protecting cells and maintaining homeostasis. When it is stimulated by pathological factors, autophagy is either disrupted or contributes to autophagic cell death. Abnormal autophagy is related to a variety of pathological disorders. Autophagy involves the following steps: autography induction, vesicle nucleation, vesicle elongation and autophagosomes formation, and retrieval and fusion between autophagosomes and lysosomes [2]. Mitochondria produce energy in the form of ATP and play important roles in cellular homeostasis, signaling, apoptosis, autophagy, and metabolism. Damaged and dysfunctional mitochondria are deleterious to cells and can even lead to various types of diseases [3,4,5,6,7,8]. Therefore, quality control of mitochondria is crucial. Autophagy is the main form of mitochondria degradation, which is a process that is called mitophagy [3,4]. Mitophagy is an autophagic response that specifically targets damaged and hence potentially cytotoxic mitochondria. Putative kinase 1 (PINK1)-Parkin axis is a well-known regulation pathway in mitophagy [9]. Furthermore, Bcl-2 interacting protein 3 (BNIP3) [10], Bcl2-like protein 13 (Bcl2-L-13) [11], and FUN14 domain containing protein1 (FUNDC1) [12] are reported to be involved in mitophagy regulation [3].

Autophagy is widely implicated in cardiac homeostasis in health and diseases, such as myocardial infarction, myocardial hypertrophy, cardiac fibrosis, cardiomyopathy, and heart failure [13,14,15,16,17]. Autophagy positively or negatively regulates myocardial infarction and myocardial hypertrophy. The modulation of autophagy processes affects cardiac function [13,18,19,20]. Cardiac fibrosis and heart failure are associated with excessive autophagy. The suppression of autophagy could attenuate fibrosis and improve heart function [16,17]. Additionally, autophagy is implicated in cardiomyopathy. It is reported that autophagy is suppressed in high-fat diet-induced obesity cardiomyopathy [15,21], while autophagy is activated in doxorubicin-induced cardiomyopathy and cardiotoxicity [22].

### 1.2. MicroRNAs

MicroRNAs (miRNAs) are small non-coding RNAs with a length of approximately 21–22 nucleotides (nt), which are distributed widely in viruses, plants, protists, and animals. They function to mediate post-transcriptional gene silencing. MiRNAs usually exhibit a high conservation level and a very low rate of evolution [23,24]. Mature miRNAs are generated via a two-step processing by Drosha and Dicer. The primary transcripts with a length of hundreds of nucleotides, which are named Pri-miRNAs, are initially generated in the nucleus. The Drasha complex cleaves pri-miRNA into pre-miRNA. Pre-miRNA is transported to the cytoplasm by Exportin-5. In the cytoplasm, pre-miRNA is processed by RNase III Dicer. Eventually, one arm is generated as the mature miRNA with a length of about 22 nt, while the other one is degraded [25,26,27,28]. The mature miRNAs are assembled into the ribonucleoprotein (RNP) complex, which are called miRNPs or miRISCs. By interacting with the 3′-untranslated region (UTR), 5′-untranslated region (UTR), or coding sequence (CDS) region of the target genes, miRNAs mediate gene silencing at the post-transcriptional or translational levels. Argonaute proteins (AGOs), which are the key components of miRNPs, compete with translation initiation factors (e.g., elf4E) by binding to their m7G cap to inhibit translation initiation. AGOs also recruit the 60S ribosomal subunit binding protein elF6 to prevent the binding of the 60S subunit, which triggers translation initiation. At the post-initiation stage, either miRNAs or AGOs become associated with polysomes to play a supposed role in elongating inhibition or the dropping off of the ribosome. Moreover, the miRNPs could recruit the CCR4-NOT complex to mediate the deadenylation of mRNAs during the translation process [29,30]. Based on their function in mediating gene silencing, miRNAs are deeply linked to numerous biological processes and related diseases [18,31,32].

## 2. MicroRNAs Regulate the Core Autophagy Signaling Cascades

**Figure 1:**
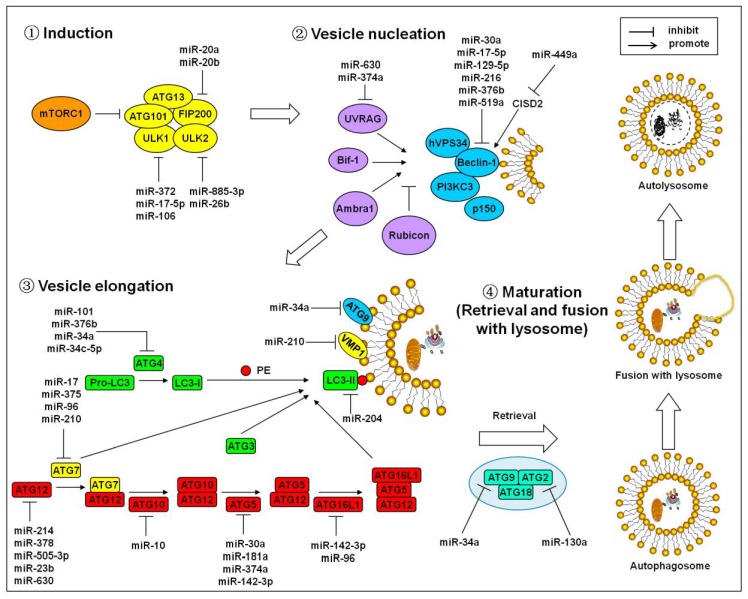
MicroRNAs regulate the core autophagy signaling cascades. Autophagy proceeds in four successive stages including induction, vesicle nucleation, vesicle elongation, and maturation. MicroRNAs are implicated in the processes. See text for detailed explanations. Arrows represent the promotion effect. T bars represent the inhibition effect.

Accumulated research has indicated that miRNAs play critical roles in the autophagy processes. Autophagy proceeds in several successive stages, including induction, vesicle nucleation, vesicle elongation, and maturation [31,33]. MiRNAs are deeply implicated in the regulation of the main autophagy signaling cascades [31,34], which usually occurs through modulating the expression of autophagy-related genes (Figure 1).

Autophagy induction is mainly regulated by the unc-51-like kinase (ULK) complex, which is composed of ULK1/2, autophagy-related gene 13 (ATG13), ATG101, and focal adhesion kinase family interacting protein with a mass of 200 kDa (FIP200). The mammalian target of rapamycin (mTOR) is one of the most important upstream regulators for the induction of autophagy. Under normal conditions, the mammalian target of rapamycin complex 1 (mTORC1) interacts with and inactivates the ULK1/2. Once exposed to autophagy-induced factors, such as myocardial ischemia, anoxia, or other myocardial damage stimuli, mTORC1 is dissociated from the ULK complex, which dephosphorylates the ULK1/2. The activated ULK1/2 subsequently phosphorylates ATG13 and TIP200, which leads to the initiation of autophagy [33,34]. It was reported that miR-372, miR-17-5p, and miR-106a inhibit autophagy by down-regulating ULK1 [35,36,37]. MiR-885-3p was found to directly target ULK2 mRNA to control the cell viability and autophagy [38]. ULK2 is suppressed by miR-26b in a targeted manner, which interrupts the autophagy initiation [39,40]. Additionally, miR-20a and miR-20b negatively regulate autophagy via targeting FIP200 [41].

Vesicle nucleation is the step that forms the double membrane-bond vesicles, which are called autophagosomes. The activation of the PI3K complex, which is composed of PI3KC3, hVPS34, Beclin-1, and p150, triggers the vesicle nucleation. In addition, several binding partners including Ambra1, Bif-1, UV irradiation resistance-associated gene (UVRAG), and Rubicon positively or negatively regulate this complex. In response to ample nutrients, the Bcl-2 family anti-apoptotic proteins interact with Beclin-1 and inhibit autophagy [31,42]. It was reported that miR-630 and miR-374a could repress the expression of UVRAG [43]. Regarding the PI3K complex, miR-30a, miR-17-5p, miR-129-5p, miR-216, miR-376b, and miR-519a prevent the autophagy process through directly targeting Beclin-1 [44,45,46,47,48]. MiR-449a indirectly promotes Beclin-1-mediated autophagy via suppressing the CDGSH iron sulfur domain 2 (CISD2) [49]. MiR-449 or miR-146a targeting Bcl-2 is also involved in autophagy regulation [50,51]. ATG12 system and LC3 system, the two ubiquitin-like protein conjugation pathways, are responsible for the vesicle elongation. In the ATG12 pathway, ATG12 interacts with ATG5 by binding ATG7 and ATG10 successively. The ATG12-ATG5 complex finally conjugates with ATG16L1 to form a large multimeric complex. In the LC3 pathway, ATG4 cleaves pro-LC3 to LC3-I. LC3-I is subsequently conjugated to phosphatidylethanolamine (PE) under the catalysis of ATG7 and E2-like ligase ATG3. Mediated by the ATG16L1-ATG5-ATG12 complex, which functions as an E3 ligase, LC3-I is processed to the phagophore membrane-bound LC3-II [31,33]. These two conjugation pathways could be regulated by microRNAs. The ATG12-mediated autophagy is targetedly inhibited by miR-214, miR-378, miR-505-3p, miR-23b, and miR-630 [43,52,53,54,55]. MiR-181a/miR-30a/miR-374a targeting ATG5 [43,56,57], miR-142-3p targeting ATG5/ATG16L [58], miR-96 targeting ATG7/ATG16L1 [59], miR-210/miR-17/miR-375 targeting ATG7 [60,61,62], and miR-20 targeting ATG10 [63] have been demonstrated to regulate autophagy. Additionally, the down-regulation of ATG4B is mediated by miR-34a/34c-5p, which suppresses the rapamycin-induced autophagy [64]. ATG4C and ATG4D are silenced by miR-376b and miR-101, respectively [48,65]. MiR-204 prevents autophagy by targeting LC3-II [66]. VMP1, a critical transmembrane protein for phagophore formation, is a direct target of miR-210 [67].

The maturation of autophagy includes retrieval and fusion with lysosomes. The process is poorly studied in mammals and humans. The retrieval step is mainly mediated by the ATG9-ATG2-AGT18 complex, which recruits lipids and proteins to the growing phagophore. Lastly, autophagosomes fuse to lysosomes, forming autolysosomes where the inner membrane of the former autophagosome and the engulfed cargo are degraded by acid hydrolases [31,68,69]. ATG9A is a direct target of miR-34a in autophagy maturation step [70]. MiR-130a interferes with the ATG9-ATG2-AGT18 complex formation via down-regulating ATG2B [71].

## 3. The Role of MicroRNAs in Cardiac Autophagy

The importance of autophagy for the preservation of cardiac homeostasis in physiological settings has been demonstrated. When they are subjected to the cardiomyocyte-specific deletion of autophagy-related gene 5 (Atg5), which is required for optimal autophagic responses, mice will develop cardiac hypertrophy, left ventricular dilatation, contractile dysfunction, and premature death. This is accompanied by a disorganized sarcomere structure, mitochondrial misalignment, and aggregation [72,73,74]. MiR-19a-3p/19b-3p inhibits autophagy in cardiomyocytes by targeting the TGF-β II-Atg 5 pathway [16]. MiR-33 reduces lipid droplet catabolism by negatively regulating Atg5 and miR-214-3p, which reduces the oxidized low-density lipoprotein-initiated autophagy by directly targeting Atg5 mRNA. This ultimately contributes to atherosclerosis [75,76]. In human aortic smooth muscle cells, the forced expression of miR-221/222 silences phosphatase and tensin homolog deleted on chromosome ten (PTEN) and subsequently activates the Akt signaling. This eventually inhibits autophagy by down-regulating the expression of LC3II and ATG5, as well as by elevating the expression of SQSTM1/p62 [77]. MiR-30b disrupts autophagy in vascular smooth muscle cells by decreasing autophagy-related genes, such as Atg5 and LC3II [78]. MiR-212 and miR-132 inhibit autophagy by negatively regulating the pro-autophagic transcription factor forkhead box O3 (FOXO3). The mice engineered to overexpress miR-212 and miR-132 in cardiomyocytes prematurely succumb because of pathological cardiac hypertrophy and heart failure [20,79]. Another critical microRNA that regulates cardiac autophagy is miR-199a. MiR-199a represses autophagy by indirectly activating the mechanistic target of rapamycin complex 1 (mTORC1), which leads to cardiac hypertrophy. In this model, tissue degeneration could be partially reversed by a potent autophagy inducer, rapamycin [14]. In addition, the silencing of miR-143 promotes the autophagy of the c-kit^+^ cardiac progenitor cells in response to oxidative stress. Autophagy-related gene 7 (Atg7) is identified as the target gene of miR-143 in the autophagy pathway [80].

Mitophagy, which is responsible for the quality control of mitochondria to match the metabolic or developmental demands, is critical for cardiac homeostasis. Mitophagy is usually executed by a series of mitochondria functional molecules, such as Bcl2 interacting protein 3 (BNIP3L, best known as NIX), FUNDC1, Parkin, PTEN-induced putative kinase 1 (PINK 1), and mitofusin 2 (MFN2) [81,82,83,84]. MicroRNAs have been found to be involved in the mitophagy flux. MiR-137 markedly inhibits mitophagy without affecting global autophagy in response to hypoxia in neurocytes. A further exploration of the mechanism shows that miR-137 targets two mitophagy receptors, FUNDC1 and NIX, and downregulates their expression. The suppression of mitophagy mediated by miR-137 could be reversed by the forced expression of FUNDC1 and NIX [85]. MiR-27a and miR-27b prevent mitophagic influx by suppressing PINK1 expression at the translational level, which subsequently decreases ubiquitin phosphorylation, Parkin translocation, and LC3 II accumulation in damaged mitochondria. Furthermore, this inhibits the lysosomal degradation of the damaged mitochondria in neurocytes [86]. MiR-181a blocks the colocalization of mitochondria and autophagosomes/lysosomes by targeting Parkin. The overexpression of miR-181a inhibits the mitochondrial uncoupling agents, which induces mitophagy without affecting global autophagy. In contrast, the knockdown of miR-181 accelerates the mitophagy in neuroblastoma cells [87]. The role of microRNAs in cardiac mitophagy has been rarely reported. In the cardiac system, miR-410 inhibits the excessive mitophagy from cardiac ischemia/reperfusion injuries by modulating the heat shock protein B1 activity via a direct interaction with the 3′-untranslated region of high-mobility group box 1 protein [88].

The degradation of damaged cells, organelles, or intracellular components by autophagy has been demonstrated to prevent the aging of cardiomyocytes. It was found that the senescent myocardium is associated with impaired autophagy. The induction of autophagy could reverse age-dependent cardiac hypertrophy and diastolic dysfunction in old mice [89,90]. During the processes, AMP-activated protein kinase (AMPK) strongly promotes an autophagy flux, which consists of unc-51 like autophagy activating kinase (ULK) 1, Beclin 1, and phosphatidylinositol 3-kinase catalytic subunit type 3 (PIK3C3) [72,91,92,93]. MiR-20a and miR-106b reduce autophagy via the suppression of the ULK1 expression in myoblasts. The cells with the knockdown of endogenous miR-20a and miR-106b exhibit a normal autophagy activity [94]. MiR-19a-3p/19b-3p targeting the Beclin 1 pathway has been demonstrated to inhibit autophagy in cardiomyocytes [16]. MiR-221/222 inhibits autophagy by indirectly regulating Beclin 1 [77]. In vascular smooth muscle cells, Beclin 1 is a target of miR-30b to mediate autophagy [78]. MiR-155 plays a beneficial role in the aging myocardium by negatively regulating the autophagy-inhibitor, mTOR [95].

In summary, autophagy and mitophagy are deeply implicated in the cardiac homeostasis processes, including heart development, compensation, and aging, which could be widely regulated by microRNAs.

## 4. MicroRNAs in Autophagy-Related Heart Diseases

**Figure 2:**
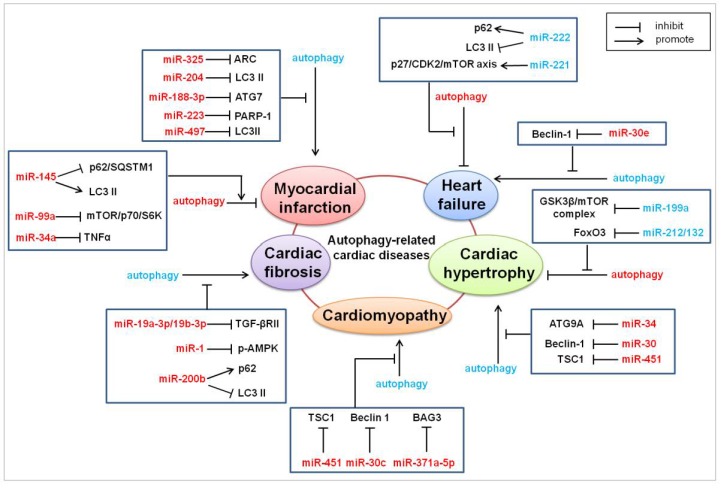
Summary of miRNAs regulating cardiac autophagy and related heart diseases. The molecular mechanisms of the miRNAs regulating the autophagy and autophagy-related heart diseases are shown in the figure. See text for detailed explanations. Arrows represent the promotion effect. T bars represent the inhibition effect. Red represents the negative regulation in diseases. Blue represents the positive regulation in diseases.

Autophagy is widely involved in cardiac physiology, and impaired autophagy or abnormal autophagy usually contribute to cardiac pathological disorders [96]. Recent research has suggested a close link between autophagy and heart diseases, including myocardial infarction [18], ischemia-reperfusion injury [66], myocardial hypertrophy [97], cardiac fibrosis [16], cardiomyopathy [98], and heart failure [17]. During the above processes, a variety of microRNAs have been demonstrated to play a critical role (Figure 2). MicroRNAs participate in regulating heart diseases through modulating the cardiac autophagic cell death. Many critical autophagy-related genes, such as ATG7, ATG9, LC3, p62, Beclin-1, AMPK, mTOR, BAG3, TNFα, and PARP-1, have been demonstrated to be direct or indirect targets of microRNAs in the autophagy-signaling pathways, which are involved in the pathogenesis of heart diseases [14,18,19,99,100,101,102,103,104,105].

### 4.1. Myocardial Infarction

Myocardial infarction (MI) is pathologically defined as myocardial cell death due to prolonged ischemia or anoxia, which is the most severe manifestation of coronary heart disease. Cardiac cell death plays a decisive role in the pathogenesis of MI, because of the terminal differentiation and loss of regenerative ability of cardiomyocytes [106]. Autophagic cell death has been demonstrated to greatly contribute to the pathogenesis of MI. As a class of important autophagy regulators, microRNAs promote or inhibit MI by mediating the autophagy pathway. 

A famous microRNA that is involved in autophagic cell death and MI is miR-325. MiR-325 is upregulated both in the cardiomyocytes treated with anoxia/reoxygenation, and in the hearts subjected to ischemia/reperfusion injury. The cardiomyocyte-specific overexpression of miR-325 potentiates excessive autophagic responses and myocardial infarct sizes, whereas the knockdown of miR-325 inhibited the autophagic cell death and MI. The apoptosis repressor with caspase recruit domain (ARC) is identified as the downstream mediator and the transcription factor, E2F1, as the upstream regulator of miR-325 in the autophagy program. A novel autophagic regulating model composed of E2F1, miR-325, and ARC in MI has been clarified in the literature [107]. The outstanding role of miR-30e in autophagic cell death and related MI has been demonstrated. MiR-30e protects hearts from MI. The cardioprotective mechanism of miR-30e has been explored. The level of miR-30e is dramatically decreased in the animal models of MI. The silencing of miR-30e significantly inhibits cellular apoptosis by modulating the apoptosis-related gene, Bax, and caspase-3, and meanwhile activates the autophagic flux and Notch1/Hes1/Akt signaling pathway. The effect of the knockdown of miR-30e on apoptosis and oxidative stress damage could be reversed significantly by autophagy inhibitor 3-methyladenine [108]. MiR-34a could inhibit autophagic cell death in the hearts that are subjected to ischemia/reperfusion via regulating tumor necrosis factor α (TNFα), thereby reducing myocardial injury [104]. MiR-145 plays a cardioprotective effect in myocardial infarction by promoting cardiac autophagy. Rabbits administrated with miR-145 mimics exhibit a significantly smaller infarct size and improved cardiac function than that of the control group upon ischemia/reperfusion injury. Further study shows that miR-145 promotes autophagic cell death in cardiomyocytes by directly targeting fibroblast growth factor receptor substrate 2 (FRS2) mRNA, and it subsequently accelerates the transition of LC3B I to II and down-regulates p62/SQSTM1, which inhibits MI [19]. MiR-223 is up-regulated in rat hearts that have undergone coronary ligation. The overexpression of miR-223 inhibits excessive cardiac autophagy. Further mechanistic study reveals that miR-223 protects cardiomyocytes from excessive autophagy via the Akt/mTOR pathway by targeting poly (ADP-ribose) polymerase 1 (PARP-1) [105]. MiR-188-3p is down-regulated in the myocardial infarction model. The forced expression of miR-188-3p suppresses autophagic cell death by targeting ATG7, which subsequently reduces the infarct sizes and improves cardiac function [18]. MiR-204 could inhibit autophagy by targeting LC3 II upon ischemia-reperfusion injury [66]. MiR-99a plays a cardioprotective role in the post-MI left ventricle remodeling by preventing cell apoptosis and increasing autophagy via an mTOR/p70/S6K pathway, which improves both the cardiac function and survival rate in a murine model of MI [109]. Myocardial miR-497 is dramatically down-regulated in the murine hearts subjected to MI, and in the cardiomyocytes subjected to hypoxia/reoxygenation. The overexpression of miR-497 induces apoptosis by targeting the anti-apoptosis gene, Bcl-2, and inhibits autophagy by targeting the autophagy gene, LC3BII, while the silencing of miR-497 exhibits the opposite effect in response to MI [110].

### 4.2. Cardiac Hypertrophy

A growing number of studies have revealed a critical pathogenic role of the altered activity of autophagy in cardiac hypertrophy. It was reported that cardiac autophagy was increased in a maladaptive manner in the hearts that were subjected to a pressure overload. However, some other studies have suggested that myocardial autophagy is insufficient, which results from a chronic pressure overload and contributes to maladaptive cardiac remodeling and heart failure [111]. MicroRNAs have been demonstrated to participate in regulating autophagic cell death in cardiac hypertrophy. In angiotensin-induced cardiac hypertrophy, miR-30 and miR-34 play an anti-hypertrophy role by preventing autophagy [97,99]. MiR-451 has been demonstrated to be involved in suppressing the autophagosome formation by targeting tuberous sclerosis complex1 (TSC1), which inhibits abnormal autophagy upon hypertrophic stimuli. By controlling the autophagy process, the ectopic overexpression of miR-451 attenuates cardiac hypertrophy, while the knockdown of miR-451 accelerates hypertrophy [112]. The MiR-212/132 family promotes pathological cardiac hypertrophy and heart failure by directly targeting the anti-hypertrophic and pro-autophagic transcription factor, FoxO3. The MiR-212/132 null mice do not frequently develop hypertrophy and heart failure, whereas the overexpression of miR-212/132 leads to the hyperactivation of pro-hypertrophic calcineurin/NFAT signaling and an impaired autophagic response in response to starvation [20]. Cardiac specific miR-199a transgenic mice suffer cardiac hypertrophy, accompanied with decreased autophagy levels. The enhancement of autophagy by the forced expression of Atg5 attenuates the hypertrophic effects of overexpression of miR-199a on cardiomyocytes. In exploring the molecular mechanism, miR-199a has been demonstrated to target the glycogen synthase kinase 3β (GSK3β)/mTOR complex signaling pathway in modulating autophagy and cardiac hypertrophy [14]. 

### 4.3. Cardiac Fibrosis 

A link between autophagy and cardiac fibroblasts has been demonstrated recently. However, the exploration on the molecular mechanism, including the role of microRNAs in autophagy-related cardiac fibrosis, is insufficient. It was reported that miR-19a-3p/19b-3p could inhibit epithelial mesenchymal transition, extracellular matrix production, and the invasion of human cardiac fibroblasts by targeting TGF-β RII. Moreover, the enhancement of autophagy rescues the inhibition effect of miR-19a-3p/19b-3p on cardiac fibroblasts. These results suggest that miR-19a-3p/19b-3p exhibits an anti-fibroblast effect through regulating cardiac autophagy [16]. MiR-1 promotes high glucose-induced cardiac fibrosis by down-regulating pro-autophagic p-AMPK [102]. MiR-200b controls cardiac fibroblast autophagy during cardiac fibrosis. The expression level of miR-200b is decreased in the cardiac fibrosis model. The MiR-200b mimic inhibits the LC3BII/I ratio, increases p62, and alleviates cardiac fibroblast autophagy, whereas the knockdown of miR-200b exhibits an opposite effect [113].

### 4.4. Cardiomyopathy 

MicroRNA-mediated autophagy is involved in the pathogenesis of several cardiomyopathy. Until now, miR-30c, miR-371a-5p, and miR-451 have been precisely demonstrated to regulate cardiac autophagy in cardiomyopathy [98,103,112]. The depletion of miR-30c enhances autophagic cell death in diabetic hearts. The overexpression of miR-30c inhibits autophagy in diabetic hearts and subsequently improves cardiac function and structure in the diabetic mice model. In exploring the molecular mechanisms, it was found that miR-30c suppressed autophagy in diabetic cardiomyopathy via targeting BECN1, through direct binding to BECN1 3′ UTR [98]. Bcl-2-associated athanogene 3 (BAG3), an autophagy pathway mediator, is a direct target of miR-371a-5p in healthy donors. Mutations and polymorphisms, including a frequent nucleotide change g2252c in the BAG3 3′-untranslated region (3′-UTR) of Takotsubo patients, leads to the loss of binding to miR-371a-5p, which probably contributes to the Takotsubo cardiomyopathy (TTC) pathogenesis [103]. MiR-451 is down-regulated in the heart tissues from hypertrophic cardiomyopathy (HCM) patients. The ectopic overexpression of miR-451 inhibits the autophagosome formation through targetedly suppressing tuberous sclerosis complex 1 (TSC1), and subsequently decreasing the size of the cardiomyocytes [112].

### 4.5. Heart Failure

Emerging evidence confirms that restoring autophagy, which improves bulk protein degradation, is proved to be beneficial in heart failure [5]. The cardioprotective effect of autophagy on heart failure could be mediated by microRNAs. MiR-221 has emerged as a representative regulator of autophagy-mediated heart failure. Research suggests that the cardiac-specific overexpression of miR-221 in mice leads to cardiac dysfunction and heart failure, and reduces the autophagic flux by inhibiting the autophagic vesicle formation in the meanwhile. Further study showed that miR-221 inhibits autophagy and promotes heart failure by modulating the p27/CDK2/mTOR axis [17]. MiR-222, which shares the same gene cluster with miR-221, exhibits a potential function in autophagic cell death and heart failure. Transgenic mice with a cardiac-specific expression of miR-222 significantly develop heart failure and are accompanied with autophagy inhibition. MiR-221 downregulates LC3 II, upregulates p62, and activates the mTOR pathway, all of which are critical autophagy regulators [100]. Another microRNA regulating cardiac autophagy in heart failure is miR-30e. MiR-30e inhibits the autophagy of cardiomyocytes by down-regulating the Beclin-1 expression, and subsequently mediates the cardioprotection of the angiotensin-converting enzyme 2 (ACE2) in the rats with Dox-induced heart failure [101].

In summary, microRNAs targeting the autophagy signaling pathways in cardiac disorders have been demonstrated by numerous evidence. The autophagic cell death mediated by microRNAs plays a critical role in the pathogenesis of heart diseases.

## 5. Conclusions and Perspectives

As outlined in this review, miRNAs function as pro- or anti-regulators in cardiac autophagy, by targeting extensive signaling pathways. This knowledge has improved our understanding of the role and mechanism of autophagy in cardiac diseases. Mitophagy is one critical part of the autophagy processes, which plays a decisive role in cardiac physiology and pathology. However, studies on the role of microRNAs in cardiac mitophagy are greatly insufficient, and thus more explorations need to be carried out in the future. Considering that, usually, one miRNA targets several genes or several miRNAs target one gene in autophagy regulation, the crosslink between the different pathways needs to be further explored. Autophagy is an evolutionarily conserved self-protective process and plays a critical role in maintaining cell homeostasis. However, excessive autophagy usually contributes to the pathogenesis of a variety of diseases, including cardiac disorders. Up until now, it has been a mystery as to how beneficial autophagy turns into a harmful autophagic cell death. With respect to MI as well as cardiac hypertrophy and heart failure, the miRNAs-regulated autophagy probably functions as the promoter or inhibitor in the pathogenesis. For instance, miR-145 plays a cardioprotective effect in MI by promoting cardiac autophagy [19], while miR-188-3p inhibits MI via inactivating the autophagy pathway [18]. MiR-451 functions as a cardiac hypertrophy inhibitor by activating autophagy [112], while the knockdown of miR-199a suppresses cardiac hypertrophy by promoting autophagic cell death [14]. MiR-221 inhibits autophagy and promotes heart failure [100], while miR-30e plays a cardioprotective role in heart failure by modulating autophagy [101]. Whether miRNA-regulated autophagy plays the role of an angel or devil in cardiac pathology, remains controversial. Cardiac cell death is the cytological basis of cardiac disorders. Apart from autophagy, several other types of cell death, such as apoptosis, are involved in the cardiac pathology. In the exploration of miRNA regulating cardiac autophagy, the apoptosis pathway is in crosslink with the autophagy pathway. For example, miR-223, miR-99a, and miR-30e regulate both the cardiac autophagy process and the apoptosis process [101,105,109]. Therefore, research focusing on the relationship of different types of cardiac cell death is necessary in the future. The important role of microRNAs in regulating cardiac autophagy has been demonstrated by accumulated evidence from studies. However, there are still many fuzzy and controversial issues that need to be explored in the future.

## Abbreviations

ACE2	angiotensin-converting enzyme 2
AGOs	argonaute proteins
AMPK	AMP- activated protein kinase
ARC	apoptosis repressor with caspase recruit domain
ATG	autophagy-related gene
BAG3	Bcl-2-associated athanogene 3
Bcl2-L-13	Bcl2-like protein 13
BNIP3	Bcl-2 interacting protein 3
BNIP3L	Bcl2 interacting protein 3 like
CDS	coding sequence
CISD2	CDGSH iron sulfur domain 2
FIP200	focal adhesion kinase family interacting protein of 200 kDa
FOXO3	forkhead box O3
FUNDC1	FUN14 domain containing protein 13
GSK3β	glycogen synthase kinase 3β
HCM	hypertrophic cardiomyopathy
MFN2	mitofusion2
miRNAs	microRNAs
mTOR	mammalian target of rapamycin
mTORC1	mTOR complex 1
PARP-1	poly (ADP-ribose) polymerase 1
PE	phosphatidylethanolamine
PIK3C3	phosphatidylinositol 3-kinase catalytic subunit type 3
PINK1	putative kinase 1
PTEN	phosphatase and tensin homolog deleted on chromosome ten
TSC1	tuberous sclerosis complex 1
TTC	Takatsubo cardiomyopathy
ULK	Unc-51-like kinase
UTR	untranslated region
UVRAG	UV irradiation resistance-associated gene
